# Participatory intervention with objectively measured physical risk factors for musculoskeletal disorders in the construction industry: study protocol for a cluster randomized controlled trial

**DOI:** 10.1186/s12891-015-0758-0

**Published:** 2015-10-16

**Authors:** Mikkel Brandt, Pascal Madeleine, Jeppe Zielinski Nguyen Ajslev, Markus D. Jakobsen, Afshin Samani, Emil Sundstrup, Pete Kines, Lars L. Andersen

**Affiliations:** National Research Centre for the Working Environment, Lersø Park Allé 105, Copenhagen, Denmark; Department of Health Science and Technology, Physical Activity and Human Performance group, SMI, Aalborg University, Fredrik Bajers vej 7, 9220 Aalborg, Denmark

**Keywords:** Participatory ergonomics, Electromyography, EMG, Video observational, Low back pain, Neck pain, Shoulder pain, Blue collar workers

## Abstract

**Background:**

There is high prevalence of back pain and neck-shoulder pain among blue collar workers in Denmark. Excessive physical exposures such as heavy lifting or working with bended or twisted back are risk factors for back pain among workers in the construction industry. Technical evaluation of awkward postures and kinematics of upper/ lower extremities (accelerometry) during work combined with the level of muscular activity (EMG) and video recordings can improve quantification of physical exposure and thereby can facilitate designing preventive strategies. Participatory ergonomics potentially increase the success of interventions aimed at reducing excessive physical exposures. The objectives of this study are to; 1) determine which work-tasks in selected job-groups involve excessive physical load of the back and shoulders during a normal working day (measured with accelerometers, EMG and video recordings). And 2) investigate whether a participatory intervention can reduce the excessive physical workloads, drawing on measurements from phase 1.

**Methods/Design:**

A two-armed parallel-group, single-blind, cluster randomized controlled trial with allocation concealment will be conducted in the Danish construction industry. Approximately 20 construction gangs (≈80 subjects) will be recruited and randomized at the cluster level (gang). We will record in situ physical workload using technical measurements (EMG, accelerometers and video recordings) during a working day before and after the intervention. Based on these measurements a physical load matrix for each worker will be developed.

The participatory intervention consist of three workshops: 1) One at baseline, involving presentation of video clips of the work-tasks with excessive physical load customized for each gang, followed by a participatory development of solutions on how to reduce excessive workloads, leading to development of an action plan on how to implement these solutions at the workplace. 2) A second workshop where the implemented solutions will be further developed and qualitatively evaluated during group discussions. 3) A final workshop at follow-up to enhance long-time organizational sustainability of the implemented solutions.

**Discussion:**

The results will provide knowledge about the level of physical exposure of the back and shoulders during specific work tasks in the construction industry, and will provide information on options to implement participatory interventions aiming at reducing excessive physical workload.

**Trial registration:**

ClinicalTrials.gov (NCT02498197), registered 29 June 2015.

## Background

Musculoskeletal disorders (MSD), especially low back pain and neck-shoulder pain, constitute a substantial problem in the working environment [1–8]. MSD can lead to physical disability, long term sickness absence [9, 10] and early retirement [11]. Prevention of MSD should have high priority as recurrent pain is common once an initial episode has occurred [12–15]. MSD have a complex etiology and are affected by individual factors as well as psychosocial and physical exposures in the working environment [16]. High physical work demands increase the risk of MSD [17, 18], e.g. heavy lifting is a risk factor for developing low back pain [19, 20], and a relationship between working with a bended or twisted back and low back pain has been demonstrated [19]. In line with this, a recent health impact assessment based on a meta-analysis using quantitative exposure information found evidence that heavy lifting during work increases the risk of low back pain [21]. This research document that lifting loads of more than 25 kg and lifting with a frequency of more than 25 lifts per day increased the annual incidence of low back pain by 4.32 % and 3.50 %, respectively [21].

Certain occupations are particularly exposed to physical risk factors. Among workers in the construction industry there is a high degree of heavy lifting combined with bending and twisting of the back [18]. A Danish survey “Work Environment & Health” from 2012 showed that 68 and 67 % of concrete workers and carpenters, respectively, work with a bended or twisted back for more than a quarter of their total working time, and 41 % and 48 %, respectively, state that the load during a typical lift exceeds 16 kg [22]. Moreover, 40-50 % of these workers report pain in the body several times a week [22].

Besides low back pain there is high prevalence of neck-shoulder pain among blue collar workers, i.e., in an Danish cohort study 29 % reported severe pain in the neck-shoulder region [23]. Accordingly, physical risk factors for neck-shoulder pain like high physical load, working with raised arms, repetitive movements [24] and working in awkward positions [25] are common in construction work.

Existing knowledge about the dose–response relationship between physical risk factors during work and MSD is almost exclusively based on self-reported data on work-load, or derived from biomechanical laboratory studies with static postures rather than dynamic lifting performed under actual working conditions [26]. Thus, obtaining technical measured information on physical work exposures during the working day may provide a more realistic picture of the physical risk factors compared with self-reports or laboratory measurements. The development of small wearable sensors has enabled measurements of physical load in situ during an entire working day [27–29]. A recent study, using this type of measurements during a working day on 200 blue-collar workers with physical strenuous work, found a positive correlation between perceived physical exertion and relative muscle load measured with surface electromyography (EMG) of the upper trapezius muscle during daily lifting tasks [27]. Furthermore, technical measurements using EMG [27], accelerometers [30], and video recordings [31, 32] or a combination of the three techniques have been used separately or in different studies to quantify physical load in workers with strenuous work [33, 34]. However, to our knowledge no intervention studies have employed these methods simultaneously to obtain detailed information about kinematics and level of muscle activity during a working day. The use of such methods to reduce excessive physical workload would likely make interventions more specific and meaningful for workers both individually and in work groups.

In Denmark, construction work is usually organized in small groups with a high degree of self-organization called construction gangs. This structure makes it possible to coordinate, collaborate and make changes in small teams, i.e. during a participatory intervention. Participatory interventions draw on workers experiences and knowledge of work to tailor the intervention to the context, culture, as well as the psychosocial and organizational conditions of the workplace. Participatory interventions that engage the workers and tailor the work to the employees’ skills and capacity, have shown to support return to work after disease due to low back pain [35]. Likewise, reduced physical work load among workers with manual handling tasks has been reported in response to a participatory intervention emphasizing the use of technical assistive devices [36]. The European Agency for Safety and Health at Work recommends employees being involved in the processes at the working site to increase motivation, and to help the employer identify solutions for solving health and safety problems [37]. Because participatory approaches have been shown to increase employee ownership and support [38], many researchers recommend participatory interventions to increase the likelihood for a successful outcome and sustainable implementation [39–42]. A recent participatory intervention based on measurements from 10 min cleaning tasks revealed a decreased cardiovascular workload, and a more variable pattern of muscle activity among cleaners [28]. The latter has previously been considered preventive for developing musculoskeletal pain [43]. However, there is a need to investigate whether such knowledge can be adapted to the construction industry, where work is changeable i.e. in terms of working tasks and conditions on varying work sites.

In this cluster randomized controlled study we implement a participatory intervention involving construction workers and managers to emphasize organizational engagement. The intervention combines technical measurements of physical work load with surveys on psychosocial and organizational conditions to develop solutions for reducing physical risk factors during construction work. The objectives of this study are to; 1) determine which work-tasks in selected job-groups involve excessive physical load of the back and shoulders during a normal working day (measured with accelerometers, EMG and video recordings). And 2) investigate whether a participatory intervention can reduce the excessive physical workloads, drawing on measurements from phase 1. We hypothesize that a participatory intervention involving both leaders and workers will lead to identifying solutions (technical, physical, organisational, etc.) to which both leaders and workers are mutually committed to (ownership), which will lead to a reduction in workload and thereby the physical risk factors for developing MSD in the construction industry.

## Methods

### Study design

A two-armed parallel-group, single-blind, cluster randomized controlled trial with allocation concealment will be conducted in a part of the Danish construction industry. Clusters will be construction gangs typically consisting of 3-5 workers. The study setting will be on the actual working sites of the workers. Data collection is expected to take place from autumn 2015 to summer 2016, and will be completed in two phases: 1) technical measurements and questionnaires to determine the tasks with excessive physical workload for groups of construction workers. 2) a cluster randomized controlled trial that investigates whether the participatory intervention can reduce the physical load of the back and shoulders during the working day.

### Ethics

According to the Helsinki Declaration all participants will be informed about the objective and content of the study before providing written informed consent to participate in the study. This information will be given in written and oral form by an experienced researcher. The study is approved by The Danish National Committee on Biomedical Research Ethics (The local ethical committee of Frederiksberg and Copenhagen; (H-3-2010-062), and registered at the Danish data Protection agency (215-57-0074) and on ClinicalTrials.gov (NCT02498197). The reporting of the study will follow the CONSORT- [44] and SPIRIT [45, 46] statements.

### Phase 1 – detection of the physical load

The physical load during the working day will be determined using technical measurements recorded simultaneously; EMG and awkward postures and kinematics of upper/lower extremities (accelerometers) which has been used in previous studies [27, 28] as well as video recordings. The equipment measuring EMG and awkward postures and kinematics will be mounted under the working clothes of workers to minimize inconvenience. Based on these measurements we will develop a load matrix for each worker in the study that will be the basis for the detection of which working tasks evoke excessive physical load. Work tasks with measurable excessive load will be identified and selected for inclusion in the workshop. During the workshop the video recordings of the work tasks with excessive physical load will be shown to the gang and their managers.

### Phase 2 – workshops and implementation

The intervention will apply a cluster randomized controlled design with technical measurements of the physical load during the working day at baseline and at 3 and 6 months follow-up. The control group will receive flyers with information on health, lifting technique and safety procedures.

**Fig. 1:**
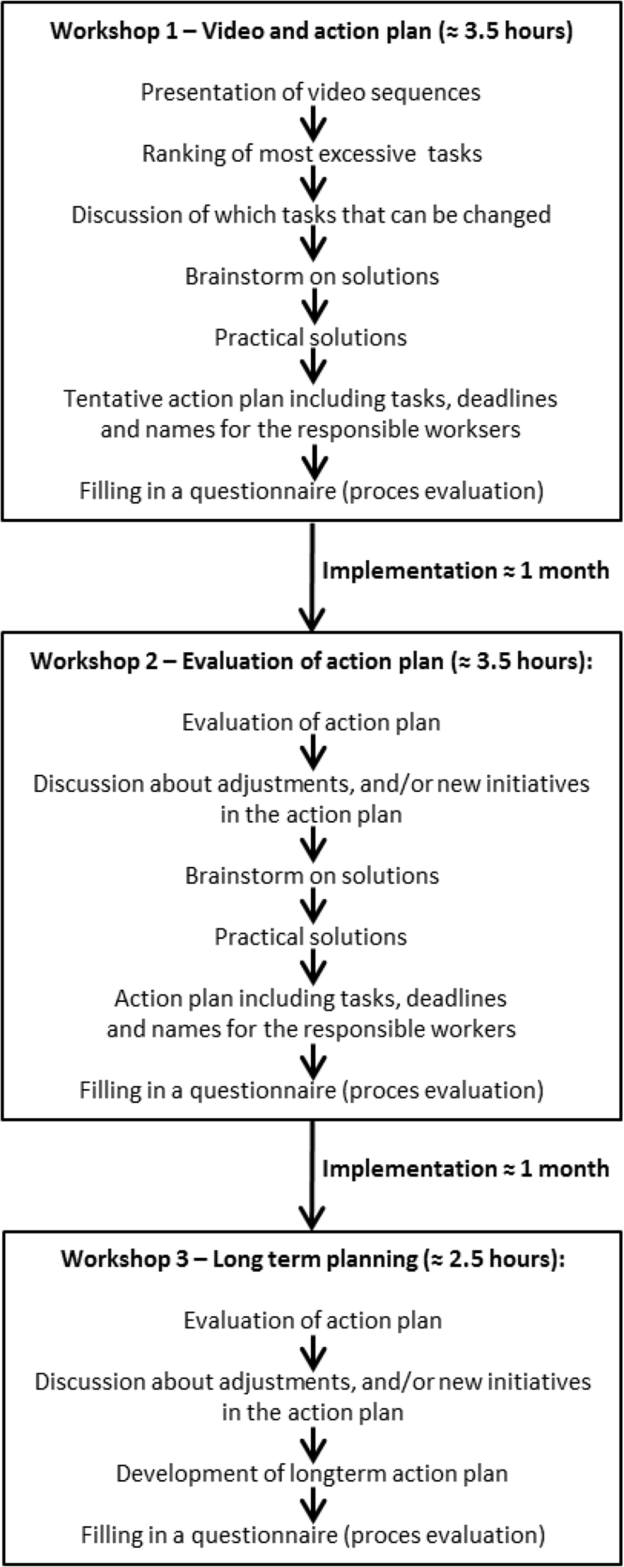
*Workshop 1:* at baseline, an opening workshop with presentation of video recordings, followed by participatory development of possible solutions on how to reduce the excessive workloads, and development of an action plan on how to implement the suggested solutions. *Workshop 2:* The solutions implemented will be qualitatively evaluated during group discussions and adjusted if necessary. *Workshop 3:* Follow-up workshop where planning on how to sustain the implemented solutions can be achieved

The intervention will consist of three workshops over a period of two months (Fig. 1). The workshops are fashioned in an experimental design inspired by methods for participation drawing on action and interactive research, emphasizing open dialogue and the inclusion of both workers and managers [47]. The inclusion of both groups is important to increase chances for organizational anchorage and continuity of solutions as construction workers often change work place and employment, and research has underlined the engagement of management as an important factor for successful organizational implementation of health and safety initiatives [48, 49].

**Fig. 2:**
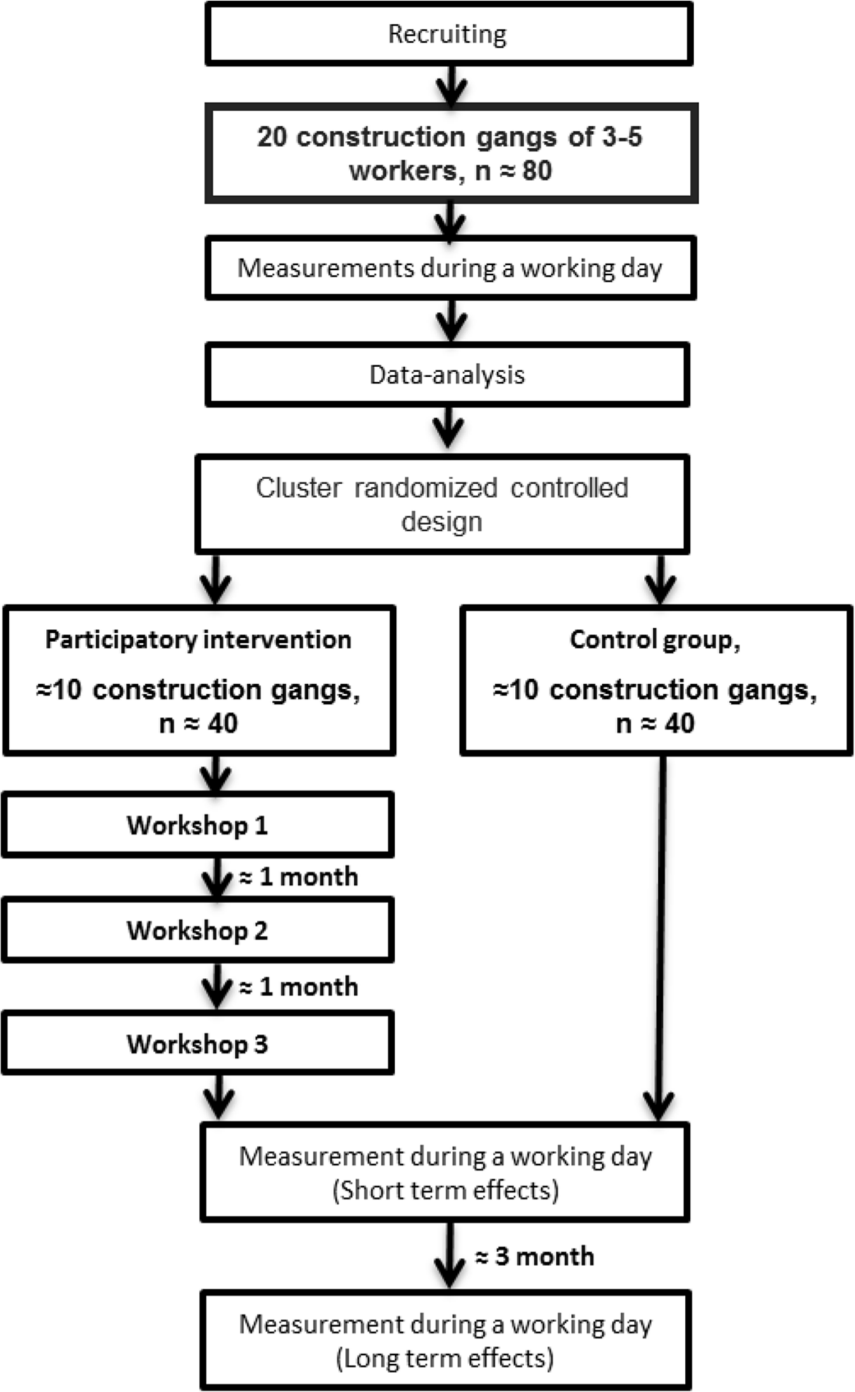
Study design and participant flow

At the baseline workshop, recordings will be presented showing work tasks with excessive physical load customized for each gang. These presentations will be followed by participatory development including individual and group-based idea-generation and discussions of possible solutions on how to reduce excessive workloads. The outcome of the first workshop is the development of an action plan on how to implement suggested solutions. At the second workshop, the solutions implemented will be qualitatively evaluated during group discussions among gang members and their leaders and adjusted with the aid of researchers and expert consultants on construction health and safety if necessary. A third workshop with gang members and their managers, where focus is on planning how long-time continuity of the implemented solutions can be achieved.

### Outcomes

The primary outcome of the present study is the change from baseline to follow-up in frequency of events with excessive physical load. Secondary outcomes are those obtained from the questionnaires, including psychosocial and organizational conditions based on the musculoskeletal pain intensity during the last week (scale 0-10), work ability index, self-efficacy and social capital (within gangs, between gangs and between gangs and their leader).

### Study population

We aim to recruit and randomize 20 construction gangs (≈80 subjects) into an intervention group or a control-group in a 1:1 allocation ratio (Fig. 2). Age and gender will reflect the distribution in the construction industry, which means that there will be a majority of men included in the study.

Inclusion criteria will be: Full time construction workers who can read and understand Danish. The type of work must include manual work that involves lifting. Exclusion criteria will be: life-threatening diseases, hypertension >160/100 mmHg, and pregnancy.

### Recruitment

The majority of participants will be recruited during 2015, and will occur in collaboration with The Danish Construction Association and consultants from The Safety and Health Preventive Service Bus for the Construction Sector. The reason for involving these institutions in the recruitment phase is that they have intimate knowledge of the construction industry and contacts to companies. The subjects will get their lost earnings covered for the time they use for participation.

### Sample size

Concerning outcome measurements, we expect EMG to be the method with lowest reliability [50]. Consequently, the power calculation is based on EMG. The primary outcome of the present study is the change from baseline to follow-up in frequency of events with excessive physical load. As the methods are new in the way that they are used simultaneously, we do not have any information on the reliability of the primary outcome, and have thus based the power calculations on closely related data. The power calculation shows that to demonstrate a reduction in 20 % in normalized EMG (nEMG) with a SD of ≈20 % in normalized EMG between individuals (based on previous research with EMG during a full working day [29]), and a type I risk of 5 % and a power on 80 % the sample size should have a minimum of 17 subject in each group. With an inflations factor of 1.5 due to the cluster randomization it will require 26 subjects in each group. For generalizability of the results we wish to include at least 10 construction gangs of 3-5 subjects in each group corresponding to approximately. 40 subjects.

### Randomization and blinding

An independent researcher will perform a block randomization on clusters of construction gangs of typically 3-5 workers. The researchers involved in the study will not be aware of the block size. Blinding of the researchers performing the measurements during the working day as well as the subjects of the study is not possible. However, as the measurements are technical, this will likely only introduce minimal bias to the results of the study. Moreover, data analysts and statisticians will be blinded to group allocation. The block randomization will be stratified for job group.

### Electromyography (EMG)

The placement of the EMG electrodes and preparation of the skin will follow the SENIAM recommendations (http://www.seniam.org). EMG signals will be recorded using bipolar EMG configuration (Blue Sensor N-00-S/25, Ambu A/S, Ballerup, Denmark) with an inter-electrode distance of 2 cm [51], bilaterally from the upper trapezius, placed at 50 % on the line from the acromion and the C7 vertebra, and from the erector spinae, placed at 2 finger width lateral from the spinous process of L1 (http://www.seniam.org). To secure an optimal EMG signal and to lower the skin-impedance the skin of the subject will be preparing with scrubbing gel (Acqua gel, Meditec, Parma, Italy) before mounting of the electrodes. The electrodes and the cables for the EMG will be fixed with tape (Fixomull stretch). The EMG signals will be normalized to maximal voluntary contractions (MVC’s) for the back and shoulders, which will be performed in a standardized procedure at the beginning and end of the working day.

As a supplement to the MVC’s, we will perform standardized reference lifts at the beginning and end of the working day. These reference lifts will be performed by lifting a weight of 30 kg (lifting limit for Danish workers) from the floor to a table five times in a standardized manner with a 10 s break between each lift.

### Awkward postures and kinematics

The kinematics of the back and shoulder will be measured using accelerometers. The accelerometers will be mounted on the upper back at the level of T1-T2 [52], lower back [53], bilateral on the upper arms below the insertion of the deltoid muscle [52], on the thigh [53] and the shank. The data will be processed in line with previous studies [30, 52].

### Video

Portable video cameras (Reveal Media, UK) will be placed on the chest of workers using a harness, thereby allowing for continuous recording during the working day. This will enable the identification of work tasks with excessive load.

### Data

EMG, kinematics and video data will be collected simultaneously to identify episodes with excessive load. The method is considered cost efficient since the researcher will not have to view 7-8 h of video manually, but only the sequences with measurements of excessive physical load (see below).

Reference lifts of 30 kg will be performed in the morning and in the afternoon, and will be used to normalize the working day EMG. Events will be defined as normalized EMG amplitudes that exceed the maximal EMG amplitude obtained during the reference lifts (averaged for morning and afternoon).

The sequences with excessive physical load will be calculated based on laboratory recordings investigating EMG activity in standardized lifting situations. The EMG activity will be calculated and used to define the cut points for excessive physical load. However, because the method is new the exact definitions of excessive physical workload will be determined during a pilot study.

Events that fulfill the criteria of excessive load will be extracted for further inspection, and an assessment of the relevance of these events will be made from the following; (i) frequency and duration (ii) physical load and (iii) practical possibility for implementation in the working context. The latter will be assessed at a workshop by the workers and managers based on the video recordings.

The workers will fill out a validated questionnaire at baseline and after the intervention period addressing psychosocial and organizational conditions at the work site [22, 54]. Answers from the baseline questionnaires will be included in the workshop interventions to increase the possibility of making practical changes in the working environment.

### Effect evaluation

The first measurement with technical measurements will serve as baseline and the follow-up measurements will be performed one week after the last workshop to measure short term effect of the intervention. Because there is approximately a month between each workshop the first follow-up measurements will correspond to a 3-month follow-up after baseline. Furthermore technical measurements will be repeated three months after the last workshop to measure the long term effect, i.e. approximately 6 months follow-up after baseline.

### Statistics

Statistical analyses will be performed using the SAS statistical software (SAS Institute, Cary, NC). The change in frequency and duration of events with excessive physical workload will be evaluated using a repeated-measures linear mixed model with *group*, *time* and *group by time* as independent variables. Participants nested within gangs will be entered as random effect to account for clustering. Analyses will be adjusted for baseline values of the number of events with excessive physical workload, gender and age. All statistical analyses will be performed in accordance with the intention-to-treat principle, i.e. including all available data in the analyses regarding actual participation or dropout. If participants do not show up for the intervention they will still be offered participation in the follow-up measurements. A P-value below 0.05 will be accepted as statistically significant. Outcomes will be reported as between-group least mean square differences with 95 % confidence intervals at follow-up.

### Adverse events

Adverse events will be registered in the questionnaires and will include musculoskeletal injuries between baseline and the last follow-up measurements.

## Discussion

This is the first participatory intervention study that identifies working conditions with excessive load among blue collar workers by measuring muscle activity (EMG), awkward postures and kinematics (accelerometry) and video recordings during a full working day, and combining these measurements with a participatory workshop based intervention that uses visualization and discussions of these specific tasks to prevent or reduce reoccurrence of the excessive loading conditions. The method has the potential to serve as a visual learning tool for the construction industry since workers can visualize tasks associated with excessive loads and customize initiatives specifically aimed at the working context. Furthermore, the detection of excessive loads will be based on tecnical biomechanical assessments performed in normal working conditions. Cluster randomization is chosen to increase adherence as well as contextual and organizational relevance and specificity by focusing on workers in their gangs, and avoiding contamination between randomized participants. As such this study will provide valuable information on how to better guide workplace initiatives aiming at reducing excessive physical work load in the construction industry.

### Strengths and limitations

A strength of the study is that real life measurements on excessive load will be used and shown as video clips to the workers during workshops as part of a participatory intervention, and thereby not limited to self-reported data and biomechanical laboratory studies. This will likely serve as an engaging, visual contextually relevant tool for both workers and managers, since they will be able to see what they are doing when exposed to excessive loads. Furthermore, the use of technical measurements will provide detailed information about the physical load in the construction industry.

The limitations of the study include the challenge to recruit construction crews performing the same work tasks during the intervention period. Construction work is often of a very varying character compared to other types of production. Due to the fact that construction work often is physically strenuous, the workers can sweat a lot during a working day, resulting in altered EMG recordings. However, we have experience from other studies [27, 28, 55], and will ensure that the electrodes are fastened in the best possible manner, and the reference lifts performed in the morning and the afternoon will provide information of possible changes in the EMG activity.

## Abbreviations

MSD: Musculoskeletal disorders
EMG: Electromyography
nEMG: Normalized electromyography
MVC: Maximal voluntary contraction
